# Neurosurgical Care of Nonpowder Firearm Injuries: A Narrative Review of the Literature

**DOI:** 10.1155/2019/4680184

**Published:** 2019-11-20

**Authors:** Yizhou Wan, Stewart Griffiths, Mario Ganau

**Affiliations:** Department of Neurosurgery, Oxford University Hospitals NHS Foundation Trust, Oxford, UK

## Abstract

**Background:**

Nonpowder firearms discharge a projectile using compressed gases. Unlike traditional firearms, there is a perception that nonpowder guns do not cause serious injury. However, intracranial injury disproportionally affects children and can cause significant neurological disabilities and mortality. Management of nonpowder firearm injuries has received little attention in the literature and presents unique surgical challenges.

**Materials and Methods:**

We conducted a narrative review of the literature of the management of nonpowder firearm injuries with particular emphasis on intracranial injury.

**Results:**

Modern nonpowder firearms have muzzle velocities which are capable of penetrating the skin, eyes, and bone. Direct intracranial injury commonly results from entrance of projectile through thinner portions of the skull. Operative intervention is needed to debride and safely explore the trajectory to remove fragments which can easily cause neurovascular injury.

**Conclusions:**

Neurosurgeons play a crucial role in managing serious nonpowder firearm injuries. A multidisciplinary team is needed to manage the direct results of penetrating injury and long-term sequalae.

## 1. Introduction

Unlike traditional firearms which use gunpowder, nonpowder firearms are designed to discharge a projectile using kinetic energy derived from compressed air and carbon dioxide or using a spring mechanism. These projectiles can be made of a variety of materials such as aluminium, lead, and plastic, and in a variety of shapes and sizes including spherical ball bearings (BB guns) and pellets.

Management of high-kinetic gunshot wounds to the cranium has been extensively reviewed. They are frequently fatal in 66–90% of cases with 71% of patients dying at the scene [1–3]. Low-kinetic cranial injuries associated with nonpowder firearms present with different aetiologies, causing different mechanisms of injury, and have been less extensively studied [4]. In the USA, nonpowder firearm injuries have long been recognised as a public health concern, particularly in children [5]. In the UK, nonpowder firearms have also been increasingly recognised as a potential cause of serious injury and death [6]. We present a review of the literature on the aetiology and neurosurgical management of nonpowder firearm cranial injuries.

## 2. Epidemiology

The incidence of nonpowder gun injuries is associated with the prevalence of nonpowder guns within the population under study. It has been estimated that by the mid-1990s in the USA, 3.2 million nonpowder firearms were sold per year causing up to 32,000 injuries per year [7, 8]. Cultural factors relating to gun use and their perception as toys may also contribute to the sale of nonpowder guns in the USA [7]. Compared to traditional firearm injuries, there are certain differences in the aetiology and populations affected. Single-centre studies from the USA have shown that nonpowder firearm injuries were more likely to be unintentional and to affect a greater proportion of Caucasian patients compared to traditional firearm injuries [9, 10]. Patients were also more frequently male and young with a mean age of 10-11 years rather than adolescents [9].

Throughout the 90s, the incidence of nonpowder gun-related injuries appears to have declined from 24.0/100,000 people in 1988 to 8.8/100,000 people in 1999 [5]. This decline may coincide with increased public awareness of the risks associated with these weapons and the increased legislation preventing their sale to minors [5]. Yet the danger from these weapons does not seem to have abated over time. In 2013, 16,259 BB or pellet gun injuries were recorded in the National Electronic Injury Surveillance System with 1237 of them estimated to occur in the head [11]. A retrospective study sampling of paediatric populations (<16 years) from three midwestern trauma centres in the USA found that nonpowder gun injuries to the head were associated with significant morbidity with 71% of patients requiring operative management and 43% of patients being left with permanent neurological deficits [11].

In the UK, there is an estimated 4 million air-powered weapons in households [12]. The Home Accident Surveillance Survey has found that there was an annual average of 1961 injuries attributable to airguns between 1989 and 1993 [12]. However, ballistics injuries may be increasing in the UK along with an increase in violent crime [13]. In one study, a large proportion (41%) of gunshot injuries to one urban trauma centre in the UK was attributable to air rifles [13]. Over time, there has been a decrease in the incidence of firearm offences along with air-weapon offences. By April 2017 and March 2018, there were 2898 air-weapon offences (30.8% of all firearm offences) recorded compared to 13822 in the period between April 2002 and March 2003 (50.7% of all firearm offences) [14]. This coincides with an increase in the bladed-weapon offences and may reflect changing trends in violent crime.

However, crime statistics do not reflect the most common method of injury which is accidental injury, particularly in children [6]. One study found in one urban UK hospital over a five-year period 73 injuries caused by air-weapons between January 1996 and June 2001. 81% of the patients were male with a median age of 15 years [6]. In cases of reported fatalities from the UK, the predominant mechanism of death is intracranial injury [6, 15, 16]. The high operative burden of managing nonpowder weapon injuries and their comparatively uncommon nature mean individual experience may be limited. Therefore, it is important for surgeons to gain a broad understanding of their management.

## 3. Medico-Legal Considerations

In the USA, regulation of nonpowder guns is applied at the state level. Federal law prevents states from prohibiting the sale of nonpowder guns but allows states to prohibit their sale to minors. Only 24 states have some form of regulation regarding the possession of nonpowder guns with only 13 states regulating the sale of nonpowder to minors. The definitions used in these laws tend to be variable with states defining minors from under 18 years to under 12 years. In addition, states vary in their definition of which nonpowder guns are firearms with some states such as New Jersey and Rhode Island classifying all nonpowder guns as firearms. The aim of this is keeping all nonpowder guns out of the hands of minors and individuals with criminal records. Other states define nonpowder guns as firearms when they exceed a certain calibre or muzzle velocity. For example, in Illinois, nonpowder guns with calibre less than 0.18 and muzzle velocity less than 700 feet/second are excluded from the definition of firearms.

The UK has some of the most stringent regulations governing airguns in the world. Apart from Northern Ireland, firearms are defined as any air pistol with a muzzle velocity of greater than 6 foot/pound or any air rifle with a muzzle velocity greater than 12 foot/pound. In Northern Ireland, nonpowder guns with a muzzle velocity greater than 0.737 foot/pound are defined as firearms [17]. Possession of these weapons requires an adult to apply for a firearm license from the police. For weapons with muzzle velocity less than these limits, the regulations vary depending on the constituent country in the UK. In Scotland, any individual over the age of 14 must apply for an Air Weapons Certificate if they use or possess an airgun from January 2017. In England and Wales, any air weapon is considered “specially dangerous” if it exceeds the limits described above and is thus regulated by firearm law.

Licenses are granted by the police to individuals who are deemed to not pose a threat to public safety and also have good reason to own a firearm. The assessment of whether an individual is fit to own forearms is the responsibility of local police forces. They may visit individual homes, check references, and request medical records from a person's primary care physician.

## 4. Mechanics of Nonpowder Weapons

All airguns propel a projectile using kinetic energy derived from compressed air or carbon dioxide. Projectiles can be made of several materials including plastic, brass, or steel and have different calibres including 0.177 (4.5 mm), 0.20 (5 mm), and 0.22 (5.5 mm) [18]. Factors which affect the degree of tissue damage include muzzle velocity, mass of the projectile, and range at which injury occurs [18].

Tissue damage caused by projectiles results from temporary cavitation or crush damage [11]. In general, the damage caused by small pellets is caused by crush of tissue in the path of the penetration. This crush is caused by shear forces generated as projectiles do not always follow a perfectly straight line to the target [19]. The ability to penetrate tissue is proportional to the kinetic energy and inversely proportional to the cross-sectional area of the projectile. Kinetic energy is proportional to the mass of the projectile and the square of the velocity of the projectile [11, 20]. Therefore, large-calibre heavier pellets are able transfer greater amounts of kinetic energy to tissue when at closer range compared to smaller lighter pellets. However, smaller projectiles can also be lethal at a close range. Forensic ballistics studies on subcalibre (0.173) steel BB pellets suggest that up to 36 mm of penetration can occur in solid bone [21]. Children are at high risk for nonpowder gun injuries not just due to the perception of airguns as toys and lack of licensing but also because paediatric patients have thinner skulls and soft tissue compared to adults [11].

Muzzle velocity is a crucial measure of the force of projectile on tissue. The primary determinants of muzzle velocity include projectile calibre, mass, propulsion system, and barrel length/width [7]. Muzzle velocities of 245 to 450 feet/sec have been found to be sufficient to cause skin penetration [7, 18]. Ocular penetration occurs at even lower muzzle velocities of 127 feet/second–246 feet/second [22, 23]. The most common calibre of pellet is 0.177 with a weight of 5.1 grains (0.33 grams) to 7.9 grains (0.5 grams) [11]. Based on this calibre and weight, skull penetration can occur in humans at muzzle velocities between 825 feet/second and 1026 feet/second [11]. The US Consumer Product Safety Commission (CPSC) estimates that over 80% of airguns sold have a muzzle velocity of greater than 350 feet/second and 50% of airguns have a muzzle velocity of between 500 and 930 feet/second [7]. Modern technologies involving CO_2_ propellant-based airguns and multiple pump action airguns can reach extremely high muzzle velocities between 400 and 450 feet/second and between 700 feet/second and 900 feet/second, respectively [18]. These muzzle velocities compare to those of conventional powder firearms, highlighting the potential lethality of these weapons. For example, the Colt 0.45 can reach a muzzle velocity of 800 feet/second [7].

## 5. Effects of Direct Intracranial Injury

Early retrospective case series describing nonpowder injuries highlighted common mechanisms of injury as important considerations for neurosurgeons. Lawrence reviewed nine cases of fatalities caused by airguns between 1956 and 1990. He found that in all but one case of cardiac injury, periorbital penetration or penetration into the thinner portions of the skull such as the pterion resulted in severe intracranial injury caused by pellet trajectories crossing the midline [18]. A 11-year review of cases at a single trauma centre in Philadelphia found that the eye, head, and neck were the most common sites of nonpowder firearm injury (41%) followed by the extremities (39%) and thorax (13%) [8]. Despite the severity of the injuries, the entrance wound may be deceptively small and easily missed [24]. The entrance site may have a small rim of abrasion but there will be no powder burns [15]. Therefore, patients with airgun injuries should be carefully evaluated in the emergency room for potential entrance sites. Relatively asymptomatic soft tissue injuries may be potentially dangerous. One case report describes a nine-year-old girl with a BB pellet entering her cheek and resting medial to the internal carotid artery [25]. Headlight with magnification may be helpful to identify the entrance site.

A recent review of nonpowder airgun injuries focusing on intracranial injuries in paediatric patients from three trauma centres found that the majority of patients were male (86%) with a mean age of nine, suffering from accidental injury in 71% of cases [11]. Skull penetration most frequently occurred in the frontal region (57%) followed by the orbital region (21%) [11]. Importantly operative intervention was required in 71% of cases including craniotomy, removal of projectile remnants, and elevation of depressed bone fragment [11]. Furthermore, the incidence of permanent neurological deficits was high including visual problems, cognitive problems, and seizures [11]. A retrospective review of paediatric airgun cases from three trauma centres in the USA found that approximately 10% of patients had intracranial injuries. Importantly, all the mortalities were from patients with intracranial injury [26].

Various case reports show that certain regions of the head are more vulnerable to airgun projectile penetration. Penetration of the thin roof of the orbital cavity is an easy route for the projectile to enter the cranial cavity [27, 28]. The entrance wound may be as smaller than 5 mm in diameter yet this disguises the severity of the intracranial damage with significant distance travelled by a pellet before stopping in the occipital lobe [27]. Along the projectile track, there can be significant damage including subarachnoid haemorrhage, subdural bleeding, and parenchymal haemorrhage [11]. It has been suggested that the passage of the projectile through the skull base can be halted by regions of relatively thicker bone such as the sella [28]. This leaves surrounding neurovascular structures such as those in the cavernous sinus vulnerable [28, 29].

The lack of cavitation damage and relatively straight projectile path means that nonpowder gun pellets are easily able to lodge into soft tissue and cause vascular laceration [30]. Case reports have shown that airgun pellets can embolise in the intracranial internal carotid artery (ICA) and travel distally to occlude the middle cerebral artery (MCA) [30, 31]. In theory, any projectile small enough to lodge into the ICA can cause distal embolization and migration, particularly in the fast-flowing arterial circulation [30]. Patients may present with hemiparesis and aphasia [30]. In an attempt to salvage neurological function, various techniques for projectile retrieval have been attempted including endovascular suction with emergency extracranial-intracranial bypass [30, 32].

These cases highlight the importance of pellet localisation. Following detailed clinical examination, the next step in management of these patients should include radiographic and computerised tomography (CT) imaging to localise projectiles, assess the degree of injury, and plan the surgical approach. There should be a low suspicion of vascular injury, especially if there is any evidence of cranial nerve palsies or entrance of the projectile involving the medial canthus or orbit. These features suggest possible involvement of the medial cranial fossa and cavernous sinus [33]. Some authors suggest that CT angiography is indicated in nearly all cases of airgun injury to the head and neck [28]. In a retrospective series of 120 patients with penetrating neck injury to the neck, CT angiography reduced the rate of negative surgical exploration by 48% [34].

Intracranial injury results in a variety of damage. Kumar et al. found in a retrospective review from three institutions that there was a wide range of overlapping pathologies including subarachnoid haemorrhage (50%), parenchymal contusion (29%), depressed bone fracture (21%), cerebral oedema (21%), intracerebral haemorrhage (21%), subdural haemorrhage (7%), intraventricular haemorrhage (7%), and pseudoaneurysm formation (7%). Amongst these patients, 71% required operative intervention [11]. Operated patients may require neurointensive care admission for neuromonitoring [35, 36]. Prevention of intensive care-related complications such as thromboembolism and delirium is necessary [37–39]. In stable patients who do not require operative intervention for intracranial pressure control, surgery may still be indicated to debride contaminated wounds and reduce the risk of late infections [28, 40–42]. Furthermore, given the proximity of projectile tracks with the skull base, duroplasty may also be needed to prevent cerebrospinal fluid (CSF) leak [43–45].

## 6. Secondary Effects of Intracranial Injury

Metallic foreign bodies which are left in situ may act as a nidus for further infections. Compared to powder-gun injuries airgun, projectiles may be more prone to infection due to their lower velocity and temperature [43]. A single-centre retrospective review over 15 years showed that long-term sequala of head and neck airgun injuries included meningitis, CSF leak, brain abscess formation, carotid-cavernous sinus fistula, intracerebral projectile migration, and projectile splitting [46].

The incidence of all infections from penetrating brain injury including soft tissue, osteomyelitis, epidural/subdural empyema, meningitis, ventriculitis, and cerebritis ranges between 5% and 23% [47]. As early as 1947, Gillingham showed that infection rates for penetrating brain injuries decreased from 25% to 5% when the length of time between injury and operative debridement was from 72 hours to 24 hours [48]. Cerebral abscesses have been reported as late as 19 months following airgun injury [16]. However, recent case series of airgun injuries have not reported any similar infections and this may be related to the use of synergistic antibiotic regimens [43]. To avoid multidrug resistance, microbiological advice and samples should always be taken where possible prior to starting treatment to allow drug rationalisation.

In wounds where there has been adequate debridement and successful removal of foreign bodies, a two-week course of antibiotics have been advocated [43]. Cairns showed in 1947 that the infectious organisms associated with penetrating intracranial wounds were related to in-driven bony fragments and from the paranasal sinuses [49]. Skin commensals such as *Staphylococcus epidermidis*, *Staphylococcus aureus*, and Gram-negative bacteria are common causative organisms [47]. Tetanus vaccination is mandatory given the risk of soil/dirt contamination of pellets [50]. Wooden pellets are especially associated with cerebral abscesses due to their porus nature offering easy bacterial culture [33]. In a series of 42 cases, Miller et al. reported that 50% of cases developed cerebral abscesses [51].

The exact incidence of postairgun injury seizures is unknown. Data from traditional powder missile injuries show that the incidence of seizures within the first 14 days is 9% and that by 24 months, the incidence can be as high as 80% [16]. In a series of 14 patients, Kumar et al. report that one patient developed epilepsy 12 years following airgun injury to the right frontal lobe [11]. In the absence of seizures, it would be prudent to follow conventional guidance and treat patients with all intracranial penetrating injuries with seven days of prophylactic antiepileptic medications.

CSF leaks can occur in 9% of patients with penetrating brain injury [47]. The incidence of CSF leaks with nonpowder projectiles entering the cranium is likely to be higher because transorbital entry is frequently associated with low-velocity projectiles [33]. In addition, low-velocity projectiles commonly enter via sinus spaces with dural breach causing communication with the intracranial compartment and acting as a nidus for infection [40]. Primary repair should be considered for any case of CSF leak associated with air-sinuses.

Vascular injury associated with airgun projectiles includes embolization of the intracranial internal carotid artery [31], pellet migration through the MCA [30], pseudoaneurysm of the anterior cerebral artery [11]. carotid-cavernous fistula [52], and possible development of dural arteriovenous fistula [53]. These injuries may be caused by skull-base fractures or shearing of the transmural vessel wall by the projectile [47]. These injuries can occur more than a week from injury, and therefore, a low threshold for angiography at diagnosis is needed [53]. Improvements in endovascular approaches such as the use of stent retrievers may improve the success rate of pellet removal. Second-line treatment requires a multidisciplinary approach given the technical challenges associated with craniotomy and embolectomy [30].

Projectile migration is a potentially serious complication [47, 54]. This may occur in the context of movement within haematoma, CSF, abscesses, and parenchyma caused pellet specific gravity and brain pulsations [54]. Studies of intracranial bullet migration show that the incidence of migration is 4.2% [55]. Copper and lead are major components of BBs, and both have been implicated in projectile migration [11]. Migration can lead to evolving neurological symptoms and can be deadly causing seizures, haemorrhage, and hydrocephalus. Importantly, migration of up to a cm has been reported three years following injury [42].

Due to potential infection and the problems associated with projectile, where possible, early surgery to explore the trajectory and remove the projectile has been recommended [28, 40, 56]. Intraoperative localisation of airgun pellets is potentially challenging. Dandy first reported using a ventriculoscope to remove a bullet from the lateral ventricle [42]. A variety of other approaches have been reported in the literature including ultrasound guidance [57], endoscopy [58], use of stereotaxis [59], intraoperative dual-plane radiography [60], and open surgery [42]. Optimal surgical strategy should be chosen balancing the risks of migration and proximity to vascular structures with the potential for iatrogenic damage.

Retained pellets may be associated with long-term complications due to the material the projectile is made from. Airgun pellets are generally made of lead (95%), tin (2.5%), and antimony (2.5%) [61]. Lead toxicity caused by retained bullets has been described [41]. Toxicity can result from levels as low as 80 *μ*g/L in children and can cause effects in multiple body systems including anaemia, renal toxicity, and encephalopathy [62].

Although lead is not ferromagnetic, some airgun pellets are made from ferromagnetic materials or coated with ferromagnetic materials such as steel [11]. Another long-term sequalae of these injuries is that future MRI scanning in these patients with retained projectiles is contraindicated as these projectiles can move in a three-tesla scanner [63]. Neurosurgeons must counsel patients and parents about this prior to discharge.

Table 1 outlines the key areas neurosurgeons must be aware of when managing patients with nonpowder gun injuries.

## 7. Conclusions

Nonpowder gun injuries are an important and underrecognised problem for surgeons. The perception of nonpowder guns as harmless recreational instruments leads to widespread societal misconceptions about their potential harms. Intracranial injuries can result in significant long-term neurological deficits and mortality. A significant proportion of patients will require operative intervention. Neurosurgeons play a crucial role in managing these patients and raising awareness of the dangers of these weapons to the public.

## Figures and Tables

**Table 1:** Summary of important considerations when managing airgun injuries.

Initial assessment
Entry wound may be inconspicuous. Assessment especially of the orbital region is needed.
Prophylactic antibiotic therapy should be instigated for all penetrating wounds.
Investigations
Low threshold for CT angiography/digital subtraction angiography.
Management
Debridement and exploration of projectile track to remove fragments where safely possible.
